# The Migration Pattern of Atrazine during the Processes of Water Freezing and Thawing

**DOI:** 10.3390/toxics10100603

**Published:** 2022-10-12

**Authors:** Yan Zhang, Chen Zhao, Aixin Yu, Wanli Zhao, Fangyun Ren, Yucan Liu

**Affiliations:** College of Civil Engineering, Yantai University, Yantai 264000, China

**Keywords:** atrazine, ice, meltwater, aquatic pollution, herbicide, distribution characteristics

## Abstract

Atrazine, one of the most commonly used herbicides in the world, is of concern because of its frequent occurrence in various water bodies and the potential threat it constitutes to ecosystems. The transport of contaminants in seasonally ice-covered lakes is an important factor affecting the under-ice water environment, and changes in phase during ice growth and melting cause redistribution of atrazine between ice and water phases. To explore the migration pattern of atrazine during freezing and thawing, laboratory simulation experiments involving freezing and thawing were carried out. The effects of ice thickness, freezing temperature, and initial concentration on the migration ability of atrazine during freezing were investigated. The results showed that the relationship between the concentration of atrazine in ice and water during freezing was ice layer < water before freezing < water layer under the ice. Atrazine tended to migrate to under-ice water during the freezing process, and the intensity of migration was positively correlated with the ice thickness, freezing temperature, and initial concentration. During the thawing phase, atrazine trapped in the ice was released into the water in large quantities in the early stages. The first 20% of meltwater concentration was significantly higher than the average concentration in ice, with the highest case being 2.75 times the average concentration in ice. The results reported in this study are a useful reference for planning possible pollution control measures on such lakes during their freeze-thaw process.

## 1. Introduction

Freezing is an important hydrological feature of lakes in high-altitude and high-latitude regions [1]. More than 50 million lakes in the world are frozen regularly [2]. The existence of an ice cover affects the light transmittance and reduces the material and energy exchange rates between the atmosphere and the water [3]. Moreover, it also reduces the rate of various biochemical reactions [4,5] and hinders a waterbody’s reoxygenation process [6,7] and photolysis reaction [8], thereby greatly weakening the self-purification capacity of a waterbody under ice [9]. The above effects reflect the prevailing viewpoint of winter as an inactive period. 

Lakes have been extensively studied in the literature, yet there are few studies on ice-covered lakes [8]. However, the lake studies that have included under-ice work strongly suggest that winter food webs and physical processes are both active and complex [7]. Aquatic ecosystems may be highly productive even when covered by snow and ice, and that winter trophic interactions can have year-round effects [8]. As the ice grows vertically downward, the solute is frozen from the lake ice and its concentration increases in the water under the lake ice [10]. Moreover, the solute frozen inside the ice is released into the water in large quantities during the early stages of thawing [11]. The transport of contaminants during freeze-thaw affects the growth and multiplication of phytoplankton and zooplankton in under-ice water [12]. Therefore, the transport of pollutants during the processes of freezing and thawing is an important factor affecting the under-ice ecology. 

Several studies have shown the migration of conventional contaminants (nutrients) during freeze-thaw. For example, Hampton et al. [8] conducted the first global quantitative integration of 101 lakes and found that the total dissolved nitrogen and total nitrogen in under-ice water during the freezing period were higher. Powers et al. [5] determined that the nitrate-nitrogen concentration in under-ice water continued to increase as the number of freezing days increased. Pieters and Lawrence [13] found that in Tailings Lake, more than 99% of the salt was excluded from ice and remained in the underlying water in winter. Zhang and Liu et al. [14,15] found that during icing, most calcium and magnesium ions migrate to water under the ice, resulting in an increase in the concentration of water under the ice. In addition, concentrations of nutrients in ice meltwater are typically not constant, especially during the ”ionic pulse” of the early melt period [16]. It was reported that the first 30% of the volume of ice melt water contains more than 90% of all impurities (salts) [17]. During thawing, dissolved organic matter trapped inside the ice was released into the aqueous environment in large quantities early in the process [11]. A few studies have shown the migration of refractory organics and emerging contaminants during the processes of water freezing and thawing [18]. Li et al. [19] studied the migration of nitrobenzene between ice and water and found that nitrobenzene tends to migrate to water under the ice during icing. Nevertheless, the migration of typical pesticides during the processes of water freezing and thawing has not been carried out. 

Atrazine is a well-known chlorotriazine herbicide widely used for weed control in agricultural crops [20]. Atrazine can migrate to the aquatic environment through rainfall-runoff, atmospheric deposition, and subsurface and groundwater leaching [21]. Due to the scale of global agricultural application, atrazine is commonly found in surface water, groundwater, sediment, soil, and the oceans [22,23,24]. Atrazine, recognized by U.S. Environmental Protection Agency (EPA) as an endocrine-disrupting compound and a possible human carcinogen, is the second most-used herbicide in the United States [25]. The use of atrazine was banned in the EU in 2004 because drinking water concentrations exceeded or were estimated to exceed permitted limits [26]. Atrazine and its metabolites can persist in water and soil for decades [26,27]. Atrazine has been reported to interfere with the physiological and biochemical systems of aquatic organisms, affecting functions such as development, reproduction and survival of aquatic biota [28]. Hence, atrazine represents a potential threat to the aquatic environment because of its high water solubility and high persistence in the environment [20].

Many organic pollutants in water can be transformed by three primary mechanisms: photodegradation, hydrolysis, and biodegradation [29,30]. However, during the freezing period, the water temperature of the lake drops, which adversely affects the degradation of atrazine [31]. Moreover, the formation of ice may increase the concentration of contaminants in the water, which are rejected into the water during the crystallization process [32]. When the lake is capped by ice, the exposure of the lake water to solar radiation is reduced, resulting in weaker photodegradation of the drug [18]. A limited number of studies have reported that conventional organic contaminants, such as organochlorine pesticides and PCBs, are observed in higher concentrations in some freeze-thaw rivers in spring due to their release from thawing ice [33]. However, there are few reports on the migration patterns of atrazine during freeze-thaw. 

Accordingly, in this study, the migration pattern of atrazine between ice and under-ice water was explored by simulating the top-down ice formation of lakes under natural conditions. The effects of ice thickness, freezing temperature and initial concentration on the migration ability of atrazine during the freezing process were investigated. Moreover, the ice obtained under different freezing conditions was thawed under natural conditions to investigate the migration pattern of atrazine during the thawing process. The results obtained herein can provide the basis for planning possible pollution control measures on ice-covered lakes during their freeze–thaw process.

## 2. Materials and Methods

### 2.1. Chemicals and Instruments

Atrazine was purchased from J&K Scientific (Beijing, China), with its purity ≥ 98.8%. Methanol and formic acid were of HPLC grade and were purchased from J&K Scientific (Beijing, China) and Kermel Chemical Reagent Co., Ltd. (Tianjin, China), respectively. A Milli-Q water purification system (MilliporeSigma, Burlington, MA, USA) was employed. Atrazine was prepared as a stock solution of 20 mg/L. The stock solution was kept in reserve at 4 °C until use.

Main testing instruments: Ultra-high-performance liquid-chromatography electrospray triple-quadrupole mass spectrometer (UPLC–ESI–MS/MS, Waters, Milford, MA, USA); an ACQUITYTM UPLC BEN C8 chromatographic column (2.1 mm × 50 mm, 1.7 μm, Waters, Milford, MA, USA). 

### 2.2. Experimental Setup

**Figure 1:**
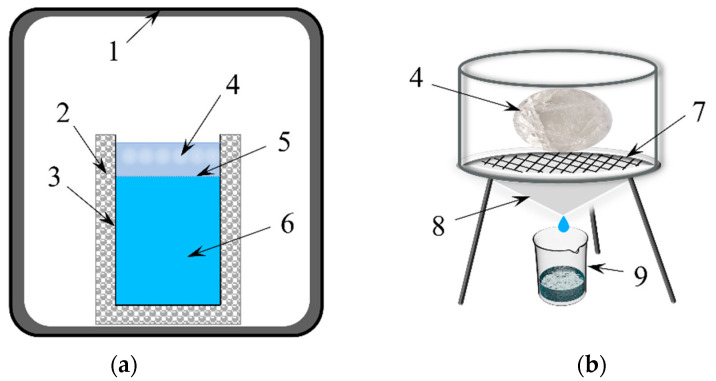
Schematic diagram of the experimental setup. (**a**) Freezing device. (**b**) Ice-melt device. (1 low temperature experiment box; 2 EPS Insulation; 3 cylindrical glass container; 4 ice layer; 5 ice/water interface; 6 water; 7 filter screen; 8 conical funnel; 9 beaker).

In order to simulate the top-down freezing process of lake water under natural freezing conditions, a self-designed unidirectional freezing simulation device was used in this study (Figure 1a). The water sample was placed in a cylindrical glass container (diameter 10 cm and height 29.5 cm). The bottom and circumference of the container were wrapped with polystyrene (EPS) insulation to ensure that the water body freezes from the top down. The experimental device was placed in a low-temperature experiment box (BC/BD-519 HEX, Haier, Qingdao, China) with temperature control. The temperature difference between the experimental box and the specified temperature did not exceed 0.5 °C. The obtained ice samples were melted in an ice melting device (Figure 1b). The ice was placed on a filter screen to melt, and at the bottom is a glass funnel and beaker to collect the ice melt water.

### 2.3. Experimental Design and Procedure

To study the distribution and migration pattern of atrazine in ice and under-ice water during lake icing, and the effects of different icing conditions on atrazine migration, the design of the experiment was as follows. Before freezing, the initial volume and height of the atrazine solution were 1.8 L and 23 cm, respectively.

(1) To explore the effect of ice thickness on the migration of atrazine, four different ice thickness conditions (3 cm, 6 cm, 9 cm, and 12 cm) were set and numbered sequentially as 1–4. Four solutions of atrazine with an initial concentration of 15 μg/L were prepared. The freezing temperature was −5 °C. Freezing ended when the ice thickness reached 3 cm, 6 cm, 9 cm, and 12 cm, respectively.
(2) To explore the effect of freezing temperature on the migration of atrazine, three different freezing temperature conditions (−5 °C, −10 °C, and −15 °C) were set and numbered sequentially as 5–7. Three solutions of atrazine with an initial concentration of 15 μg/L were prepared. Freezing ended when the ice thickness reached 9 cm.
(3) To explore the effect of initial concentration on the migration pattern of atrazine, five different concentrations (5 μg/L, 15 μg/L, 25 μg/L, 35 μg/L, and 45 μg/L) of atrazine solutions were prepared and numbered sequentially as 8–12. The freezing temperature was −5 °C. Freezing ended when the ice thickness reached 9 cm.

The atrazine solutions were frozen in the freezing device. After freezing, the freezing device was removed from the low-temperature experiment box and the ice and water were separated. The ice melted in the ice melting device and the ambient temperature of the melting was 22 °C. After the ice melted, the concentrations of atrazine in the collected meltwater and under-ice water were measured by UPLC-ESI-MS/MS.

To study the migration pattern of atrazine in ice during melting, the freezing experiments numbered 1–12 were repeated and the 12 ice cubes obtained were subjected to the melting experiment. The ambient temperature of the melting was 22 °C. The melt water of each ice block was divided into five batches in turn (Melt 1, Melt 2, Melt 3, Melt 4 and Melt 5), and the mass of each batch of melt water was 20% of the total mass of the ice. Melt 1 was the first 20%-fraction of the ice-melt water, Melt 2 was the second 20%-fraction, etc. The freezing and melting experiments were repeated three times.

### 2.4. UPLC Method Parameters and Precision

Chromatographic conditions: The mobile phase was methanol (A) and 0.1% formic acid in water (B). The flow rate was 0.2 mL/min. The column temperature was 35 °C and the room temperature for sampling was 15 °C. The injection volume was 5 μL. 

Mass spectrometry conditions: Electrospray ion source (ESI) as positive ion mode acquisition; multiple reaction monitoring (MRM) mode. The capillary voltage and cone voltages were 3.3 kV and 35 V, respectively. The ion source temperature and desolvation (nitrogen) temperature were 120 °C and 350 °C, respectively. The desolvation flow rate and the cone (nitrogen) flow rate were 500 L/h and 30 L/h, respectively. The MRM parameters are listed in Table 1.

In the concentration range of 0~100 μg/L, this method exhibited a good linearity for detecting atrazine (Y = 1337.6X + 6314.4; R^2^ = 0.995). At the low (2.5 μg/L), medium (10 μg/L), and high (25 μg/L) levels of atrazine, the recovery rate of the standard addition was between 96% and 112%, and the relative standard deviation (RSD) of the determination was between 0.39% and 0.92%. Hence, the method was accurate and reliable.

### 2.5. Date Analysis

The distribution coefficient (K) was used to express the migration ability of atrazine during freezing, as defined by Equation (1):

(1) $$K=\frac{C_{i}}{C_{w}}$$

where C_i_ is the atrazine concentration in the ice (μg/L) and C_w_ is the atrazine concentration in the water under ice (μg/L). The value of K can be between 0 and 1. A value of K = 0 indicates that the ice formed is completely pure, and all of the atrazine migrates into the water under the ice. A value of K = 1 indicates that the ice and water have the same concentration of atrazine, and no migration occurs during the process. The lower the K value, the stronger the ability of atrazine to migrate to water under the ice.

The melt-out rate (R_m_) is used to indicate the effect of atrazine release from ice during melting, as defined by Equation (2):

(2) $$R_{m}=\frac{m_{e}}{M_{A}}$$

where m_e_ is the mass of atrazine in each meltwater (μg) and M_A_ is the total mass of atrazine wrapped in ice (μg).

## 3. Results

### 3.1. Effect of Ice Thickness on the Migration of Atrazine

**Figure 2:**
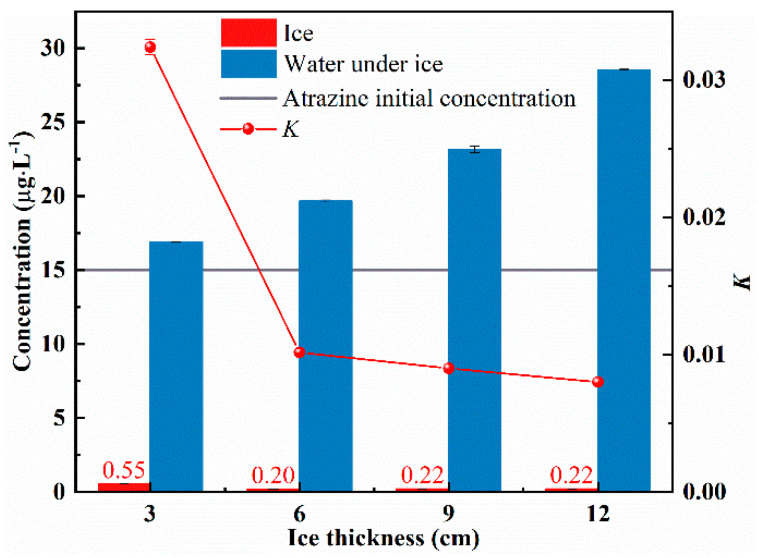
Ice-water distribution of atrazine at different ice thicknesses.

Ice and water samples were collected at different ice thicknesses (3 cm, 6 cm, 9 cm, and 12 cm) and atrazine concentrations were measured. The distribution of atrazine in ice and under-ice water at different ice thicknesses is shown in Figure 2. Atrazine concentrations in ice were 0.037 (for 3 cm), 0.013 (for 6 cm), 0.014 (for 9 cm), and 0.015 (for 12 cm) times of the initial concentration, respectively, while the atrazine concentrations in under-ice water were 1.128 (for 3 cm), 1.312 (for 6 cm), 1.544 (for 9 cm), and 1.903 (for 12 cm) times of the initial concentration, respectively. The concentration of atrazine in ice was substantially lower than the concentration of raw water before icing and the concentration of under-ice water after icing. This implies that atrazine tends to migrate more to the under-ice water. Therefore, the atrazine was concentrated in the under-ice water. Additionally, what emerges from the results here is that the thicker the ice cover, the higher the concentration of atrazine in the under-ice water. The values of the K were 0.032 (for 3 cm), 0.010 (for 6 cm), 0.009 (for 9 cm) and 0.008 (for 12 cm). As the ice grows, the value of K is decreasing. This indicates that the ability of atrazine to migrate from the ice to the water is enhanced.

### 3.2. Effect of Freezing Temperature on the Migration of Atrazine

**Figure 3:**
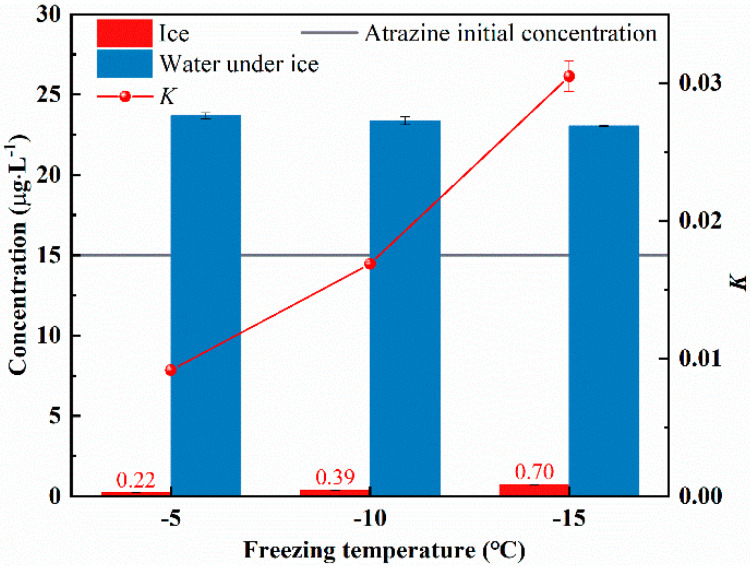
Ice-water distribution of atrazine at different freezing temperatures.

Ice and water samples were collected at different freezing temperatures (−5 °C, −10 °C, and −15 °C) and atrazine concentrations were measured. The distribution of atrazine in ice and under-ice water at different freezing temperatures is shown in Figure 3. Atrazine concentrations in ice were 0.014 (for −5 °C), 0.026 (for −10 °C), and 0.047 (for −15 °C) times of the initial concentration, respectively, while the atrazine concentrations in under-ice water were 1.579 (for −5 °C), 1.558 (for −10 °C), and 1.537 (for −15 °C) times the initial concentration, respectively. What emerges from the results here is that the lower freezing temperature causes an increase in the concentration of atrazine in the ice and a decrease in the concentration of atrazine in the water. The values of the K were 0.009 (for −5 °C), 0.017 (for −10 °C), and 0.030 (for −15 °C). Hence, the value of K tends to increase with decreasing freezing temperature. This means that the ability of atrazine to migrate from ice to water decreases as the freezing temperature decreases.

### 3.3. Effect of Initial Concentration on the Migration of Atrazine

**Figure 4:**
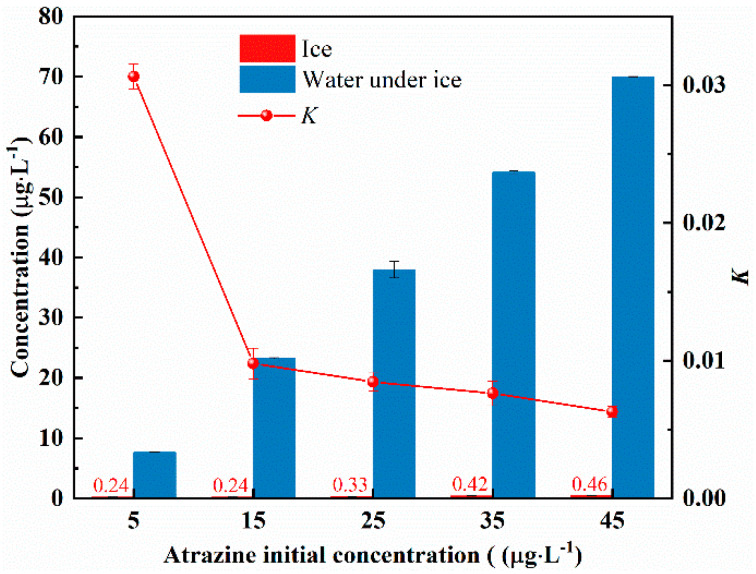
Ice-water distribution of atrazine at different initial concentrations.

Ice and water samples were collected at different initial concentrations (5 μg/L, 15 μg/L, 25 μg/L, 35 μg/L, and 45 μg/L) and atrazine concentrations were measured. The distribution of atrazine in ice and under-ice water at different initial concentrations is shown in Figure 4. Atrazine concentrations in ice were 0.047 (for 5 μg/L), 0.016 (for 15 μg/L), 0.013 (for 25 μg/L), 0.012 (for 35 μg/L), and 0.010 (for 45 μg/L) times of the initial concentration, which were significantly lower than that before freezing. Atrazine concentrations in under-ice water were increased to 1.535 (for 5 μg/L), 1.551 (for 15 μg/L), 1.520 (for 25 μg/L), 1.547 (for 35 μg/L), and 1.555 (for 45 μg/L) times of the initial concentration. The values of the K were 0.031 (for 5 μg/L), 0.010 (for 15 μg/L), 0.008 (for 25 μg/L), 0.007 (for 35 μg/L), and 0.006 (for 45 μg/L). The value of K decreases as the initial concentration increases. These results suggest that the ability of atrazine to migrate from the ice to the water improves with an increasing initial concentration in the solution.

### 3.4. Release Pattern of Atrazine during Ice Melting Process

**Figure 5:**
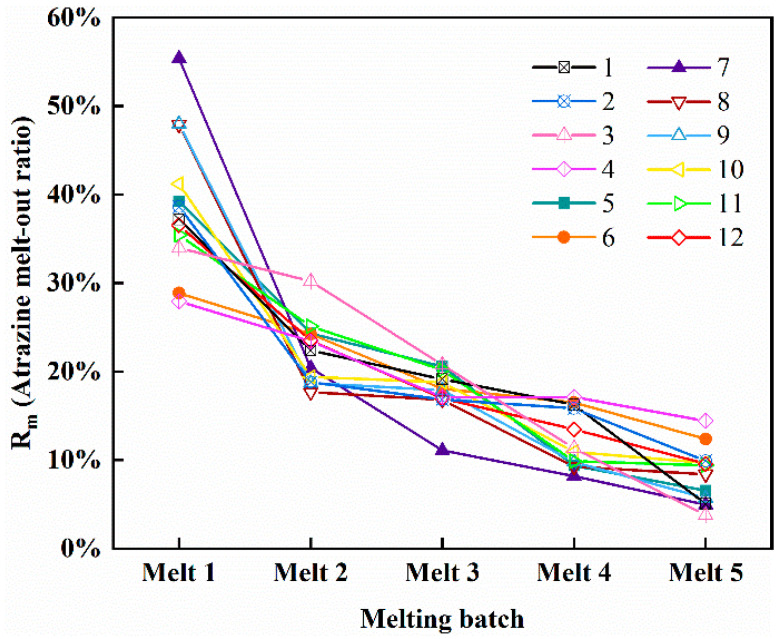
Melt-out ratio (R_m_) of atrazine during melting.

The ice bodies obtained under different freezing conditions were melted. The average concentration of meltwater and the change in meltwater concentration during melting are shown in Table 2. As the melting proceeded, the concentration of atrazine in the meltwater became lower and lower. The first 20% of the ice meltwater (Melt 1) concentrations were all higher than the average concentration of atrazine in the ice. From Melt 3 onwards, the concentration of atrazine in ice meltwater was lower than the average concentration of atrazine in ice. The concentration of atrazine in Melt 1 of the ice with the highest level of contamination was 2.75 times higher than the average concentration in the ice. Figure 5 shows the melt-out rate of atrazine during the melting process. R_m_ = 20% means that the mass transfer rate of atrazine is consistent with the melting rate of the ice. It can be seen that the R_m_ of atrazine in the first 20% fraction of the ice meltwater significantly exceeded 20%, which indicates that atrazine tends to be released from the ice when it first starts to thaw. The amount of atrazine in ice meltwater at the beginning of melting is mainly influenced by the degree of contamination of the ice itself. However, there are differences in the ice obtained under the same conditions. The structure of the ice also affects the release of atrazine in the early stages of melting. This may be responsible for the difference in the amount of atrazine released by ice bodies with similar average concentrations at the beginning of melting. Overall, atrazine remaining in the ice was released from the ice in large amounts during the early stages of melting. As melting progresses, the unmelted ice becomes more ”pure”.

## 4. Discussion

### 4.1. Migration Mechanism of Atrazine between Ice and Water during Freezing

The migration process of atrazine between ice and water could be explained by the formation and growth of ice crystals. The temperature of the upper surface of the solution decreased after the solution was placed at a low temperature. Liquid water becomes metastable when the temperature falls below the equilibrium freezing point. Water crystallization is accompanied by probabilistic nucleation events [34]. When the supercooling overcome the free energy barrier, the supercooling disappears, and nucleation occurs due to the latent heat released during the short stage of the recalescence [34,35]. These ice nuclei grow dendritic ice crystals, which then gradually form larger and more complete ice. These ice nuclei and dendrites are pure water (solid) [36]. During the growth of ice, the solutes are spontaneously repelled and retained in the water phase due to the orderly association of water molecules to structure ice crystals [37,38]. Therefore, the concentration of atrazine at the ice-water interface is higher [39]. Driven by the concentration gradient, vertical convection is generated in the water beneath the ice [40]. In straightforward terms, atrazine is repelled by the ice and migrates toward the bottom (i.e., the lower concentration region) during the crystallization of water molecules [41,42]. In addition, as the energy of the system decreases, the energy difference between the solute and the water and ice molecules drives the migration of the solute from the unstable ice phase to the stable water phase [14,43].

**Figure 6:**
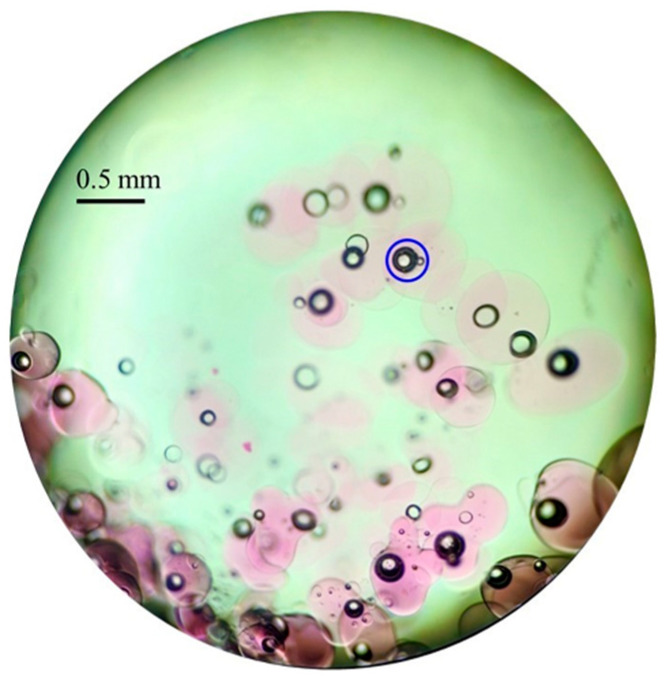
Microscopic view of ice structure (The purple color is KMnO_4_ and the blue circles indicate the formation of a trapped bubble during the freezing process).

However, there is a limit to the ability of ice crystals to repel impurities during the growth of ice. We observed that a small amount of atrazine is retained within the ice, especially during the early stages of freezing. The reason for this is that when the supercooling of the solution is broken, the system releases heat and randomly generates ice nuclei. Atrazine is trapped in the ice due to the rapid growth of ice nuclei and dendritic ice crystals. This is similar to the process of salt pack trapping during seawater freezing [44]. During the freezing process, the ice front has a large number of dendritic ice crystals that keep crystallizing. The atrazine repelled by the ice is mainly distributed in the gaps and spreads continuously downward [45]. When adjacent ice crystals combine together, at this point water and atrazine become encapsulated in the ice, and the atrazine cannot diffuse and is eventually trapped in the ice. This is similar to the formation process of air bubbles in ice [34]. Our research group has previously performed a freeze concentration test of a chromogenic substance (KMnO_4_) [46]. Moreover, it was found that KMnO_4_ was concentrated in water under the ice, and a small amount of KMnO_4_ was kept in the ice. Figure 6 shows the distribution of KMnO_4_ in the ice as observed through the microscope. The unescaped KMnO_4_ was mainly distributed near the bubbles. This may be related to the growth of ice crystals. Atrazine may have a similar distribution and migration pattern to KMnO_4_.

### 4.2. Influence of Various Factors on the Migration Capacity of Atrazine

The migration ability of atrazine in ice-water phases is affected by factors including ice thickness, freezing temperature, and atrazine initial concentration [42]. During the freezing process, as temperature decreases, ice crystals gradually grow and form ice cover. The presence of ice cover weakens the heat exchange between the solution and the surface gas [10]. With the continuous increase in ice thickness, the growth rate of ice decreases. The slow ice growth rate facilitates the diffusion of atrazine at the ice-water interface to the bottom [14]. In this study, the increase in ice thickness diminishes the ability of ice crystals to trap atrazine, thereby reducing the average atrazine concentration in the ice and enhancing the ability of atrazine to migrate from the ice to the under-ice water.

The freezing temperature determines the freezing rate, nuclear density, shape, and size of ice crystals [34]. As the freezing temperature decreases, more crystal nuclei and ice crystal branches are produced in the ice body, the freezing rate of ice increases, and the water molecules move to the ice–water interface faster. Once this speed exceeds the speed of atrazine moving to the interface, atrazine will be trapped by the ice [47]. At the same time, as the freezing temperature reduces, the solution area for the release of latent heat increases and ice crystals grow in dendrites. In addition, more branches are produced on the trunk, and atrazine is more likely to be ”trapped” by dendritic ice crystals or branch gaps [45,48]. The purity of ice crystals then decreases and the *K* value increases with decreasing temperature; hence, the migration ability of atrazine from ice to water under the ice is weakened.

For atrazine solutions with different initial concentrations, the freezing point decreases as the initial concentration of the solution increases [49]. The stability of the ice–water interface also decreases and the formation of dendritic ice crystals with higher branches can capture atrazine more easily, thus reducing the purity of ice crystals [50]. On the other hand, an increased initial concentration of the solution also increases the number of potential crystal nuclei in the solution. Moreover, the frequency and energy of collisions between crystal nuclei increase, the probability of secondary nucleation increases, ice crystal growth accelerates, and purity decreases [51]. The atrazine concentration in ice increases as the solution concentration increases. However, although the amount of captured atrazine in ice increases, it is more advantageous for atrazine to migrate to water under ice during the freezing process, the increase in atrazine concentration in ice at various concentrations was less than the migration [42]. Therefore, the K value of atrazine decreases with an increase in the initial concentration, and the migration ability of atrazine from ice to water under ice increases.

### 4.3. Mechanism of Atrazine Release from Ice during Melting

When the ambient temperature is above the freezing point, the surface of the ice begins to melt. The rate of melting is determined by the heat transfer from the environment to the ice surface and corresponds to the droplets’ growth rate at the melting interface [52]. Droplet growth is a continuous process in which atrazine can attach to the resulting droplets, which is defined as ”solute elution” [53]. The effect of elution is influenced by the rate of melting. In this study, ice melting was performed at room temperature and therefore melted faster compared to the actual lake ice melting. At lower ambient temperatures, the initial melting out of solutes would be more pronounced [54]. Atrazine is relatively concentrated in ice encapsulated in salt cells or channels, rather than uniformly distributed within the ice body. Because of this, melting causes the otherwise closed channels to be opened and atrazine is released in large quantities in the early stages. The more contaminated the ice body is, the more material is melted out in the pre-melting period. This is consistent with the observation of Htira et al. [55] that the purity achieved by the ice after melting is independent of the level of contamination in the ice before melting.

## 5. Conclusions

In this study, the distribution and migration of atrazine in ice and water phases during freezing and thawing under different freezing conditions were investigated. The main conclusions are as follows.

(1) Freezing causes redistribution of atrazine in both ice and water phases, and its concentration relationship is expressed as ice < water before freezing < water under the ice. Atrazine continuously migrated from the ice to the water under the ice during the freezing process, which reminded us that we need to pay more attention to the quality of under-ice water in the natural water body during the freezing period.
(2) During the initial stages of thawing, atrazine trapped in the ice exhibits a massive release into the water. Moreover, as thawing continued, the concentration of atrazine in the meltwater became lower and lower the unmelted ice becomes purer. In areas where the water environment is prone to freezing in winter, the water environment may deteriorate instantaneously due to the concentrated release of atrazine in the early stages of ice thawing.
(3) The K value decreased with increasing ice thickness, freezing temperature, and atrazine initial concentration. The migration ability of atrazine was positively correlated with ice thickness, freezing temperature, and atrazine initial concentration. Atrazine has an extremely low distribution coefficient (K = 0.006) when a solution with an initial concentration of 45 μg/L is frozen at −10 °C to a thickness of 9 cm. The migration ability of atrazine was strongest under this condition.

## Figures and Tables

**Table 1:** MRM method parameters of atrazine.

Compound	Mass to Charge Ratio *m*/*z*		Collision Energy
	Parent Ion	Daughter Ion	/eV
		95.8	25
Atrazine	215	103.73	28
		173.9	17

**Table 2:** Atrazine concentrations in ice meltwater: averaged value and partial values in each of 20%-fractions during melting.

Number	Average Concentration (μg/L)	Melt 1 (μg/L)	Melt 2 (μg/L)	Melt 3 (μg/L)	Melt 4 (μg/L)	Melt 5 (μg/L)
1	0.55	1.03	0.62	0.53	0.45	0.14
2	0.20	0.39	0.19	0.17	0.16	0.10
3	0.21	0.36	0.32	0.22	0.12	0.04
4	0.22	0.31	0.26	0.19	0.19	0.16
5	0.21	0.42	0.26	0.22	0.10	0.07
6	0.39	0.56	0.47	0.35	0.32	0.24
7	0.69	1.90	0.70	0.38	0.28	0.17
8	0.24	0.57	0.21	0.20	0.11	0.10
9	0.25	0.59	0.23	0.22	0.12	0.07
10	0.33	0.68	0.32	0.31	0.18	0.16
11	0.45	0.79	0.56	0.45	0.22	0.21
12	0.46	0.84	0.54	0.39	0.31	0.22

## Data Availability

The data presented in this study are available on request from the corresponding author. The data are not publicly available due to privacy reasons.
